# Assessing the Impact of the COVID-19 Pandemic on Emergency Department Use for Patients Undergoing Cancer-Directed Surgeries

**DOI:** 10.3390/curroncol29030153

**Published:** 2022-03-10

**Authors:** Antoine Eskander, Qing Li, Jiayue Yu, Julie Hallet, Natalie Coburn, Anna Dare, Kelvin K. W. Chan, Simron Singh, Ambica Parmar, Craig C. Earle, Lauren Lapointe-Shaw, Monika K. Krzyzanowska, Timothy P. Hanna, Antonio Finelli, Alexander V. Louie, Nicole Look-Hong, Jonathan C. Irish, Ian Witterick, Alyson Mahar, David R. Urbach, Danny Enepekides, Rinku Sutradhar, on behalf of the Pandemic—Ontario Collaborative in Cancer Research (POCCR)

**Affiliations:** 1 ICES, Toronto, ON M4N 3M5, Canada; qing.li@ices.on.ca (Q.L.); julie.hallet@sunnybrook.ca (J.H.); natalie.coburn@sunnybrook.ca (N.C.); anna.dare@gmail.com (A.D.); kelvin.chan@sunnybrook.ca (K.K.W.C.); simron.singh@sunnybrook.ca (S.S.); ambica.parmar@mail.utoronto.ca (A.P.); craig.earle@ices.on.ca (C.C.E.); lauren.lapointe-shaw@uhn.ca (L.L.-S.); monika.krzyzanowska@uhn.ca (M.K.K.); tim.hanna@kingstonhsc.ca (T.P.H.); antonio.finelli@uhn.ca (A.F.); nicole.lookhong@sunnybrook.ca (N.L.-H.); rinku.sutradhar@ices.on.ca (R.S.); 2 Institute of Health Policy, Management, and Evaluation, University of Toronto, Toronto, ON M5T 3M6, Canada; 3 Department of Otolaryngology—Head and Neck Surgery, University of Toronto, Toronto, ON M5S 1A8, Canada; jonathan.irish@uhn.ca (J.C.I.); ian.witterick@sinaihealth.ca (I.W.); danny.enepekides@sunnybrook.ca (D.E.); 4 Division of Biostatistics, Dalla Lana School of Public Health, University of Toronto, Toronto, ON M5T 3M7, Canada; jiayue.edith.yu@outlook.com; 5 Department of Surgery, University of Toronto, Toronto, ON M5T 1P5, Canada; 6 Department of Medicine, Division of Medical Oncology, University of Toronto, Toronto, ON M5S 3H2, Canada; 7 Department of Medicine, University of Toronto, Toronto, ON M5S 3H2, Canada; 8 Division of Radiation Oncology, Queen’s University, Kingston, ON K7L 5P9, Canada; 9 Department of Radiation Oncology, University of Toronto, Toronto, ON M5T 1P5, Canada; alexander.louie@sunnybrook.ca; 10 Department of Community Health Sciences, University of Manitoba, Winnipeg, MB R3E 0W3, Canada; alyson.mahar@umanitoba.ca; 11 Department of Surgery, Women’s College Hospital, Toronto, ON M5S 1B2, Canada; david.urbach@wchospital.ca

**Keywords:** cancer, surgery, health services research, quality of care, COVID-19, emergency department

## Abstract

Emergency department (ED) use is a concern for surgery patients, physicians and health administrators particularly during a pandemic. The objective of this study was to assess the impact of the pandemic on ED use following cancer-directed surgeries. This is a retrospective cohort study of patients undergoing cancer-directed surgeries comparing ED use from 7 January 2018 to 14 March 2020 (pre-pandemic) and 15 March 2020 to 27 June 2020 (pandemic) in Ontario, Canada. Logistic regression models were used to (1) determine the association between pandemic vs. pre-pandemic periods and the odds of an ED visit within 30 days after discharge from hospital for surgery and (2) to assess the odds of an ED visit being of high acuity (level 1 and 2 as per the Canadian Triage and Acuity Scale). Of our cohort of 499,008 cancer-directed surgeries, 468,879 occurred during the pre-pandemic period and 30,129 occurred during the pandemic period. Even though there was a substantial decrease in the general population ED rates, after covariate adjustment, there was no significant decrease in ED use among surgical patients (OR 1.002, 95% CI 0.957–1.048). However, the adjusted odds of an ED visit being of high acuity was 23% higher among surgeries occurring during the pandemic (OR 1.23, 95% CI 1.14–1.33). Although ED visits in the general population decreased substantially during the pandemic, the rate of ED visits did not decrease among those receiving cancer-directed surgery. Moreover, those presenting in the ED post-operatively during the pandemic had significantly higher levels of acuity.

## 1. Introduction

The COVID-19 pandemic has disrupted cancer care. Cancer patients are a vulnerable population with increased risk of morbidity and mortality from COVID-19 [1,2]. To help reduce the risk of exposure and transmission, the healthcare sector pivoted toward minimizing in-person visits, including for cancer care [3,4]. Not surprisingly, in the early days of the pandemic, there were drops as large as 89% in the use of the emergency departments (ED), and patients presenting in-person had much higher acuity problems [5].

ED use is an important quality metric and outcome of surgical care and a unique concern for patients, physicians and health administrators given the unique challenges in navigating healthcare during a pandemic. Cancer surgery patients are at particularly high-risk of requiring acute assessment or interventions and seeing their care disrupted by pandemic-related changes. Indeed, delays and cancellations in cancer surgery associated with the ramp down of surgical services to create capacity for COVID-19 care during the pandemic may lead to advanced presentations with high symptom burden or complications requiring urgent care. In addition, increased use of virtual care and lack of access to physical clinics for post-surgical follow-up may translate into increased reliance on the ED for management of post-operative events [6]. This is particularly crucial knowing that delays in surgery for cancer patients, such as the ones observed during the pandemic, lead to increased morbidity and mortality [7,8]. Furthermore, decreased access to surgical beds may have necessitated shorter lengths of stay which may have increased ED use. However, little is known about ED use among patients undergoing surgery for cancer and how this may be compounded by advanced presentations and ability to access post-operative care.

The objective of this study was to assess the impact of the COVID-19 pandemic on ED use following cancer-directed surgeries, with a view to better understand healthcare utilization in a resource limited environment and to inform innovative structures of care for future waves of the pandemic. We hypothesized that there would be lower use of the ED in the general population but increased use in patients who had undergone cancer-directed surgeries.

## 2. Materials and Methods

### 2.1. Study Design and Population

We conducted a retrospective cohort study using population-wide administrative databases from the province of Ontario, Canada. All patients undergoing cancer-directed surgeries from 7 January 2018 to 27 June 2020 were identified using the Canadian Institute for Health Information’s Discharge Abstract Database (CIHI-DAD) and CIHI-Same Day Surgery (CIHI-SDS) database, as previously published by our group [8,9]. CIHI includes procedure data but also diagnostic information through the integration of ICD10-CA codes. Only institutions with complete reporting on cancer-directed surgeries in both the pre-pandemic (on or prior to 14 March 2020) and pandemic (15 March 2020–27 June 2020) periods were included. Patients without a valid Ontario Health Insurance Plan (OHIP) card were excluded from the study, as were those who did not have a complete follow-up window of 30 days following discharge from hospital. We also excluded minor colposcopy, colonoscopy and sigmoidoscopy biopsy type procedures.

### 2.2. Data Sources

We linked health administrative databases held at ICES (previously known as the Institute for Clinical Evaluative Sciences). The Canadian Institute for Health Information’s National Ambulatory Care Reporting System (CIHI-NACRS) database was used to capture emergency department visits and corresponding information on acuity scores from the Canadian Triage and Acuity Scale (CTAS). Date of death and demographic characteristics were obtained from the Registered Persons Database (RPDB) [10]. Immigration status was determined using the Immigration, Refugees and Citizenship Canada (IRCC) database, where those with a “date of landing record” were immigrants. Information on comorbidities, type of cancer-directed surgery (as previously reported by our group) [8], its level of urgency and the corresponding hospital’s academic status were retrieved from CIHI-DAD and/or CIHI-SDS. The Ontario Marginalization Index (ONMARG), which focuses on both health and social well-being, was used to capture elements of marginalization, including material deprivation, residential instability, ethnic concentration and dependency [11]. More specifically, comorbidity was assessed using the Elixhauser comorbidity classification system with a 5 year look back window; if patients did not have a hospitalization that was categorized separately from those with comorbidity data, 0, 1, 2 or 3+. Cancer surgery type was classified by body subsite based on the type of surgical intervention that was captured through CIHI-DAD and CIHI-SDS; central nervous system tumors, colorectal, endocrine, esophagus, genitourinary (not including prostate), prostate, gynecological, head and neck, hepatobiliary, lung, gastric, sarcoma, melanoma and skin [8,9]. These datasets were linked using unique encoded identifiers and analyzed at ICES.

### 2.3. Main Exposure, Outcome and Other Covariates

The primary exposure was the period during which the cancer-directed surgery occurred: pre-pandemic (on or prior to 14 March 2020) vs. pandemic (on or after March 15, 2020; ramp down initiation for elective surgery). The primary outcome was the odds of experiencing an ED visit within 30 days following discharge from surgery. Characteristics measured at the time of cancer-directed surgery included: patient factors—age, sex, rurality, neighborhood income quintile, region of residence based on Local Health Integrated Networks (LHINs; 14 geographic regions in Ontario), immigration status (immigrant vs. not), marginalization quintiles, Elixhauser comorbidity level (no hospitalization, 0, 1, 2, 3+) based on a 5 year look back window prior to the time of surgery [12,13]; surgical factors—type of cancer-directed surgery, urgency of surgery (urgent—either arrived to hospital via ambulance or were admitted through the emergency department vs. not); and hospital factors—type of corresponding hospital (teaching vs. not).

### 2.4. Statistical Analysis

Analyses were conducted at the level of cancer-directed surgeries. The distributions of baseline characteristics were assessed among individuals undergoing surgery in the pre-pandemic period compared to those in the pandemic period. Due to the large cohort size, standardized differences (rather than *p*-values) were used to establish whether covariate distributions were balanced between periods; a standardized difference <0.1 indicated balance.

A funnel plot was produced to illustrate the crude rates of ED visits within 30 days following discharge from cancer-directed surgery in the pandemic period compared to the same corresponding weeks in the prior year, across region of residence. Each LHIN is represented by a dot in the funnel plot, where the x-coordinate provides the number of cancer-directed surgeries occurring in the LHIN and the y-coordinate provides the corresponding percentage that end up in ED within 30 days following discharge. The horizontal line illustrates the average ED rate, overall, and it is surrounded by the corresponding 95% confidence limits. These limits represent the expected bound around the average proportion for varying population sizes [14]. This was designed to compare the ED visit rate visually and statistically by LHIN pre- and during COVID-19 following discharge from hospital for a cancer-directed surgical resection. We further examined the pattern of ED rates in our cancer-directed surgery cohort against the pattern of ED rates among all Ontarians. Rates during the pandemic period were compared to the rates during the same corresponding weeks in the prior year, across region of residence (LHIN). These comparisons were illustrated using a histogram, where the y-axis on the left-hand side and corresponding bars reflect the ED rates in our cohort, and the y-axis on the right-hand side and corresponding overlaid points provide the ED rates in the entire province.

Univariate and multivariable logistic regression models were used to determine the association between period of cancer-directed surgery (pandemic vs. pre-pandemic) and the odds of an ED visit within 30 days after discharge. A generalized estimating equations approach was incorporated to account for possible correlation of surgeries undergone in the same institution. The regression models adjusted for all covariates listed above based on an a priori analysis plan. In addition to the main effects model, 2-way interactions between period of cancer-directed surgery and various covariates were also explored. This was performed to assess if the impact of the pandemic on the odds of ED varied by levels of socioeconomic status, comorbidity and other covariates.

Lastly, among all cancer-directed surgeries that had an ED visit within 30 days following discharge, we examined the association between the period of cancer-directed surgery (pandemic vs. pre-pandemic) and the odds of the ED visit being of high acuity. Acuity was determined using CTAS, a 5-level scale where each level is determined based on a patient’s need for medical interventions with level 1 being resuscitation for conditions that are life or limb threatening, while level 5 is non-urgent, typically chronic, problems [15]. High acuity was defined as resuscitation (most ill group), emergent or urgent levels (levels 1–3); low acuity was defined as semi-urgent or nonurgent (least ill group) levels (levels 4–5) [16]. All statistical analyses were conducted using SAS Enterprise Guide 7.15 (SAS Institute, Inc., Cary, NC, USA).

### 2.5. Ethical Standards

This study involved secondary data analyses only and was thus exempt from requiring REB approval because ICES is a designated “45.1 entity” under the Personal Health Information Protection Act (PHIPA) enabling the use of personal health information.

## 3. Results

Our population-based cohort consisted of 499,008 cancer-directed surgeries, of which 468,879 occurred during the pre-pandemic period and 30,129 occurred during the pandemic period. Table 1 compares the distributions of baseline characteristics between surgeries in the pre- and peri-periods. The mean age (SD) of patients undergoing surgery was 57.9 (17.2) in the pandemic period compared to 56.2 (16.9) in the pre-pandemic period. During the pandemic period, higher percentages of surgeries related to breast, colorectal and genitourinary cancers were found, along with a lower percentage of melanoma-related surgeries. In the pandemic period, 27.9% of surgeries were urgent in nature, whereas 14.8% were considered urgent in the pre-pandemic period. No differences were seen in the distributions of socioeconomic measures such as rurality and material deprivation between periods.

Surgery by cancer type did not differ in the two cohorts for central nervous system tumors, endocrine, esophagus, genitourinary (not including prostrate), prostate, gynecological, head and neck, hepatobiliary, lung, gastric, sarcoma and non-melanoma skin. It did however increase for colorectal (from 16.5% of cases to 21.4%; standardized difference 0.12), breast (from 6.8 to 9.8%; standardized difference 0.11) and genitourinary (not including prostate; from 6.9 to 10.1%; standardized difference 0.12) and decrease for melanoma (from 7.9 to 4.9%; standardized difference 0.12). There were no differences by region (14 local health integration networks) of residence.

**Figure 1:**
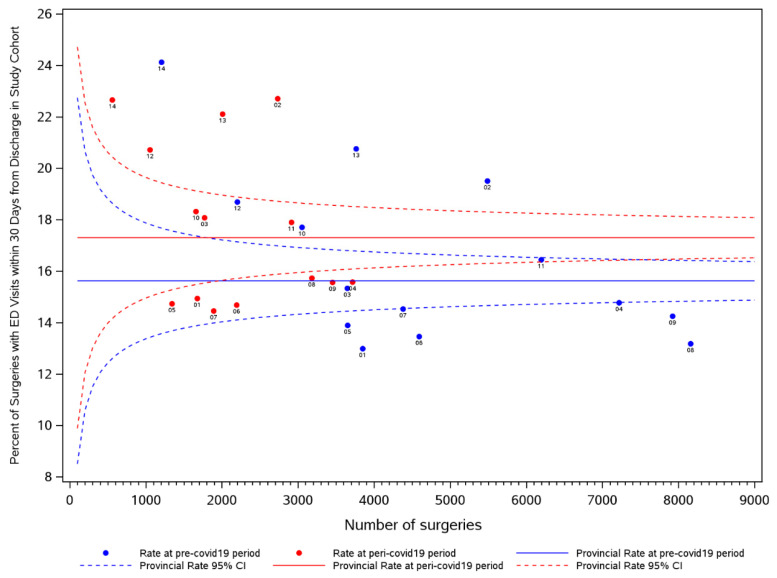
Funnel plot illustrating variation in ED rates following cancer-directed surgeries in the pre- and peri-periods, across LHINs. **Legend**: Each LHIN is represented by a dot in the funnel plot, where the x-coordinate provides the number of cancer-directed surgeries occurring in the LHIN and the y-coordinate provides the corresponding percentage that ends up in ED within 30 days following discharge. The horizontal line illustrates the average ED rate, overall, and it is surrounded by the corresponding 95% confidence limits. These limits represent the expected bound around the average proportion for varying population sizes [14].

**Figure 2:**
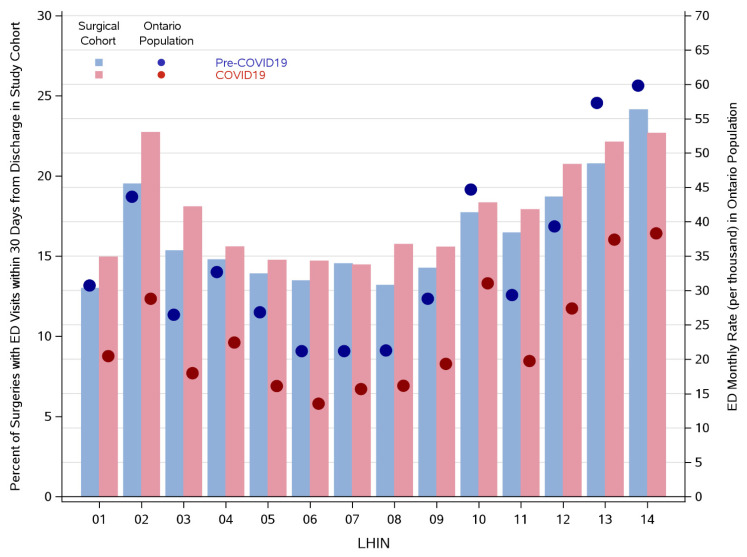
Histogram illustrating ED rates within our cohort of cancer-directed surgeries compared with ED rates in the general provincial population during the pre- and peri-periods, across LHINs. **Legend:** The y-axis on the left-hand side and corresponding bars reflects the ED rates in our surgical cohort, while the y-axis on the right-hand side and corresponding overlaid points provide the ED rates in the entire province.

The variation in ED rates following cancer-directed surgeries in the pandemic period compared to the same corresponding weeks in the prior year is seen by the funnel plot in Figure 1. Based on these crude (unadjusted) proportions, the mean rate of ED visits post discharge was higher during the pandemic period (solid red line) compared to the pre-pandemic period (solid blue line). Most regions had an increase in 30-day ED rates post discharge during the pandemic period compared to the pre-pandemic period. Figure 2 depicts the comparison of 30-day post discharge ED rates after cancer-directed surgery (bars) to ED rates (for any reason) in the general population (overlaid dots). While the rates of 30-day ED visits after cancer-surgery increased from the pre- to COVID-19 period, with absolute differences varying from 0.1 to 3.2% depending on region, the average monthly ED visits rate for the general population decreased, with absolute differences varying from 5.0 to 21.1 per 1000. This trend was observed across all regions. Taking LHIN 2 again as an example, although the proportion of surgeries with an ED visit within 30 days following discharge increased from 19.5% during the pre-period to 22.7% during the peri-period, the average monthly ED rate (for any reason) among all Ontarians decreased from 43.5 per 1000 to 29.0 per 1000, respectively; similar trends were seen in most LHINs. In the general population, pre-pandemic and pandemic ED visits led to inpatient admission in 11.3 and 13.2%, respectively, while in the surgical cohort, admissions were required in 19.3 and 25.1%, respectively.

**Figure 3:**
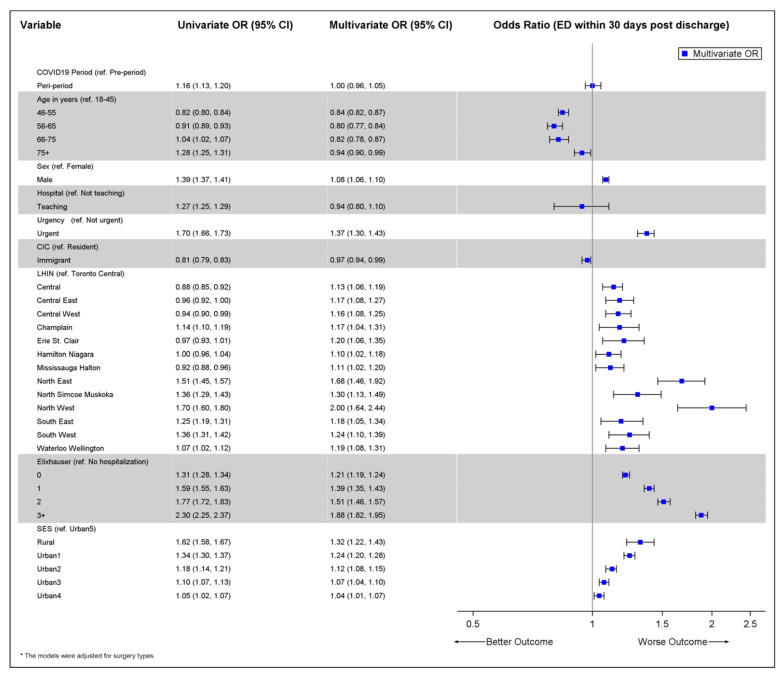
Forest plot illustrating the associations between characteristics and the odds of an ED visit within 30 days after discharge from cancer-directed surgery (among entire cohort of cancer-directed surgeries).

The associations between the period of cancer-directed surgery (pandemic vs. pre-pandemic) and odds of 30-day ED visits after cancer-directed surgery are illustrated by the forest plot in Figure 3. On unadjusted (univariable) analysis, there was a significant association between the period of cancer-directed surgery and odds of an ED visit within 30 days (OR 1.16, 95% CI 1.12–1.20). However, this association no longer holds after multivariable adjustment and adjustment for possible correlation within institutions (OR 1.002, 95% CI 0.957–1.048, *p*-value 0.93). One of the strongest associations was between urgency of surgery and odds of ED. Surgeries considered urgent had 38% higher odds of experiencing an ED visit compared to non-urgent surgeries (OR 1.38, 95% CI 1.31–1.45, *p*-value < 0.01). There was a clear gradient in the association between comorbidity and ED visit, where those with a larger number of comorbidities had higher odds of ED visit. There were no significant interactions found between period of cancer-directed surgery and other characteristics (data not shown).

**Figure 4:**
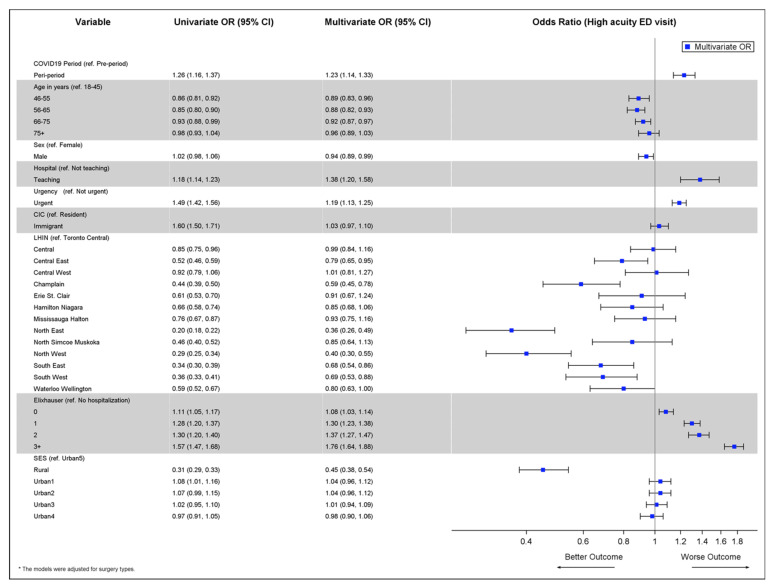
Forest plot illustrating the associations between characteristics and the odds of the ED visit being of high acuity (among cohort of cancer-directed surgeries that resulted in an ED visit within 30 days post discharge).

The association between period of cancer-directed surgery (pandemic vs. pre-pandemic) and the odds of the ED visit being of high acuity in the sub-group of patients who had an ED visit after surgery are illustrated in Figure 4. After adjusting for other variables and possible institution-level correlation, the odds of an ED visit being of high acuity was 23% higher among surgeries occurring during the pandemic compared to those occurring prior to the pandemic (OR 1.23, 95% CI 1.14–1.33). There was again a clear gradient in the association between comorbidity and ED acuity, where those with a larger number of comorbidities had higher odds of a high acuity ED visit.

## 4. Discussion

This population-based retrospective cohort study demonstrated a drop in ED visits from the pre- to the pandemic period for the general population, with absolute differences varying from 5.0 to 21.1 per 1000. After adjustment, the surgical cohort ED rates post-operatively were stable but with higher acuity in the pandemic period. During the pandemic period, higher percentages of surgeries related to breast, colorectal and genitourinary cancers took place, with a lower percentage of melanoma-related surgeries. Urgent procedures comprised 27.9% in the pandemic period, whereas 14.8% were considered urgent in the pre-pandemic period.

In a publicly funded healthcare system, we have previously demonstrated equally equitable access to surgical care during the pandemic compared to pre-pandemic, particularly early on during the first wave [8]. That initial work however also uncovered a higher proportion of “urgent” surgery in the pandemic period compared to pre-pandemic. Therefore, as the delivery of perioperative care changed to adapt to the demands of the pandemic, for example, by limiting in-person visits, the patterns of surgical care also changed with a potential for more acute perioperative needs. It was crucial to better understand the repercussions of those changes on the outcomes of care as this may provide information about gaps in care that could be prevented in future waves of the pandemic but also during the next few years as surgical systems recover from the pandemic backlog in both surgical procedures and cancer diagnoses. It also can provide important insight into how our system functions under challenging situations. The cohort studied in this analysis is an extension of the one we had initially reported on [8].

We observed an increase in unadjusted 30-day ED rates post discharge during the pandemic period compared to the pre-pandemic period amongst surgical patients despite a drop in the monthly ED rate (for any reason) among the entire population for the same period, which was consistent across regions. One of the strongest associations was between urgency of surgery and odds of ED visit; surgeries considered urgent had 38% higher odds of experiencing an ED visit compared to non-urgent surgeries. Rural patients were more likely to use the ED peri-operatively (Figure 3); however, when they presented to the ED, they were more likely to present with lower acuity problems (Figure 4) suggesting that they were using the ED for assessment complaints that could be handled in other ways if they were available to them. This is an important point as it describes how the ED in regions of lower physician density may be used and speaks to access to non-urgent physician care both pre- and during the pandemic. After adjusting for other variables and possible institution-level correlation, the odds of an ED visit being of high acuity were 23% higher among surgeries occurring during the pandemic compared to those occurring prior to the pandemic, which is foreseeable given our previous observation of more urgent higher risk surgery being completed during the pandemic period.

The drop in ED use in the general population observed in our study is in keeping with other jurisdictions [5]. This is not particularly surprising given the messaging around minimizing in-person visits to help mitigate exposure and transmission, particularly during the early pandemic period [3,4]. Even though there was a substantial decrease in overall ED rates in the general population, there was no significant decrease in ED use among surgical patients. However, presenting to the ED with higher acuity scores was more common in the pandemic period. This is likely related to patients presenting in a deferred fashion with more complex issues which is known to be associated with higher complication rates, both immediate and delayed [17,18]. One such study from New Zealand specifically addressed patients with appendicitis, cholecystitis and diverticulitis, all of which are common general surgery emergency department diagnosed illnesses and as such it is not surprising that with decreased ED use there were fewer presentations, in a delayed fashion with increased complications and length of stay [17,18]. Similarly, a UK study demonstrated the same concerns with emergency general surgery admissions [17,18]. However, there is conflicting evidence regarding this, with other studies suggesting that COVID-19 related delays for surgical patients did not lead to higher morbidity and mortality [19]. This particular study from Germany looked at all elective general, thoracic and vascular surgeries at two centers and did not identify any additional risks from short delays in surgery related to the pandemic [19]. Our study confirms that in our jurisdiction there is at the very least no decrease in ED use among those receiving cancer-directed surgeries despite low ED use in the general population. This is not likely related to COVID-19 itself but other concerns that patients have, given that nosocomial COVID-19 transmission has been extremely low with modern day infection prevention and control processes [20]. Furthermore, most patients that presented to the ED (11–25%) did not require admission, which suggests patients may be presenting with concerns that could be managed on an outpatient basis. We hypothesize that decreased access to in-person outpatient care may have contributed to ED use, although this requires further study. While telehealth may be an appropriate initial evaluation for post-operative complications [21], alternate pathways are required to ensure patients can bypass the ED if an evaluation or admission is required. Increased access to homecare, after hour physician and nursing access lines and real-time symptom monitoring could all improve access to care during these circumstances and possibly decrease emergency department use. However, this requires additional investigation through prospective studies.

These data must be interpreted in the context of the study design. This study did not account for the COVID-19 status of the patients as this data was not readily available for all patients in our cohort at the time of study completion. This makes it hard to ascertain whether ED use was related to COVID-19 related complications. Nonetheless, COVID-19 testing for surgical patients was routine and patients who were COVID-19 positive pre-operatively were delayed. It is therefore unlikely that nosocomial or delayed COVID-19 diagnoses is what accounted for the stable ED use. Furthermore, the exact cause of admission was not extracted. This could provide further insight into patterns of preventable causes and may lead to multifaceted interventions to help curb emergency department use. Nonetheless, this is the first and largest study to assess this question at a population-based level. The study is strengthened by its large sample size, a focus on cancer-directed surgeries and a high capture of post-operative events.

## 5. Conclusions

In conclusion, although the rate of overall ED in the general population decreased substantially during the COVID-19 pandemic compared to the pre-pandemic period, the rate of ED visits did not significantly decrease among those receiving cancer-directed surgery. Moreover, those presenting in ED post-operatively during the COVID-19 pandemic period had significantly higher levels of acuity compared to those in the pre-pandemic period. Further work is required to investigate the possible causes for these findings and identify potential gaps in care for patients during a pandemic whilst accounting for COVID-19 status during the observation trajectory.

## Figures and Tables

**Table 1:** Distributions of sociodemographic and hospital characteristics for surgeries performed during the pre- and COVID-19 periods.

Variable	Pre-COVID-19 * (*N* = 65,309)	COVID-19 (*N* = 30,129)	Standardized Difference ^a^
Age (Mean ± SD)	56.2 ± 16.9	57.9 ± 17.2	**0.10**
Female	40,341 (61.8%)	17,488 (58.0%)	0.08
Rural Score (Rio 2008)—0–9	42,447 (65.8%)	19,203 (64.6%)	0.03
10–30	11,996 (18.6%)	5689 (19.2%)	0.01
31–50	7176 (11.1%)	3435 (11.6%)	0.01
51–70	1962 (3.0%)	929 (3.1%)	0
71+ (more rural)	902 (1.4%)	456 (1.5%)	0.01
Immigrant	9583 (14.7%)	3813 (12.7%)	0.06
Elixhauser Grouping ^b^—0	9380 (14.4%)	4262 (14.1%)	0.01
1	5664 (8.7%)	3089 (10.3%)	0.05
2	3426 (5.2%)	1955 (6.5%)	0.05
3+	4572 (7.0%)	2689 (8.9%)	0.07
No Hospitalization	42,267 (64.7%)	18,134 (60.2%)	0.09
Material Deprivation Quintile 1—Least Deprived	14,252 (22.0%)	6602 (22.1%)	0
2	13,916 (21.5%)	6108 (20.5%)	0.03
3	12,377 (19.1%)	5763 (19.3%)	0
4	12,018 (18.6%)	5626 (18.9%)	0.01
5—Most Deprived	12,146 (18.8%)	5734 (19.2%)	0.01
Inpatient Surgery ^c^	28,080 (43.0%)	17,779 (59.0%)	**0.32**
Non-Teaching Hospital Status	47,657 (73.0%)	20,384 (67.7%)	**0.12**
Urgent ^d^	9451 (14.5%)	8410 (27.9%)	**0.33**

* same period was used (2019, 10 March 2019–22 June 2019, pre-COVID-19; 2020, 15 March 2020 to 27 June 2020, COVID-19). ^a^ Standardized difference of > 0.1 (**bold**) was used to indicate a clinically and statistically significant imbalance in the distributions of the characteristics. ^b^ The Elixhauser comorbidity grouping was calculated using a 5 year look back window in administrative data for any hospitalization. This is a well validated approach to assess comorbidities using administrative data. Although “no hospitalization” is grouped with 0 in many prior studies, these categories were separated here to provide additional information on comorbidity variation for the reader. ^c^ This variable is a measure of procedures that were performed on an inpatient basis as opposed to same day surgery (same day discharge or discharge from a short-stay unit after 1 night overnight stay). ^d^ Patients treated urgently either arrived at hospital via ambulance or were admitted through the emergency department.

## References

[1] Dai M., Liu D., Liu M., Zhou F., Li G., Chen Z., Zhang Z., You H., Wu M., Zheng Q. (2020). Patients with cancer appear more vulnerable to SARS-CoV-2: A multicenter study during the COVID-19 outbreak. Cancer Discov..

[2] Yang F., Shi S., Zhu J., Shi J., Dai K., Chen X. (2020). Clinical characteristics and outcomes of cancer patients with COVID-19. J. Med. Virol..

[3] Al-Shamsi H.O., Alhazzani W., Alhuraiji A., Coomes E.A., Chemaly R.F., Almuhanna M., Wolff R.A., Ibrahim N.K., Chua M.L., Hotte S.J. (2020). A Practical Approach to the Management of Cancer Patients During the Novel Coronavirus Disease 2019 (COVID-19) Pandemic: An International Collaborative Group. Oncologist.

[4] Sun H., Liu K., Li M., Tang S., Monte A.A., Wang J., Nie S., Rui Q., Liu W., Qin H. (2020). The influence of coronavirus disease 2019 on emergency department visits in Nanjing, China: A multicentre cross-sectional study. Am. J. Emerg. Med..

[5] Butt A.A., Azad A.M., Kartha A.B., Masoodi N.A., Bertollini R., Abou-Samra A.-B. (2020). Volume and Acuity of Emergency Department Visits Prior To and After COVID-19. J. Emerg. Med..

[6] Glazier R.H., Green M.E., Wu F.C., Frymire E., Kopp A., Kiran T. (2021). Shifts in office and virtual primary care during the early COVID-19 pandemic in Ontario, Canada. Can. Med. Assoc. J..

[7] Hanna T.P., King W.D., Thibodeau S., Jalink M., Paulin G.A., Harvey-Jones E., O’Sullivan D.E., Booth C.M., Sullivan R., Aggarwal A. (2020). Mortality due to cancer treatment delay: Systematic review and meta-analysis. BMJ.

[8] Eskander A., Li Q., Hallet J., Coburn N., Hanna T.P., Irish J., Sutradhar R. (2021). Access to Cancer Surgery in a Universal Health Care System During the COVID-19 Pandemic. JAMA Netw. Open.

[9] Juurlink D., Preyra C., Croxford R., Chong A., Austin P., Tu J., Laupacis A. (2006). Canadian Institute for Health Information Discharge Abstract Database: A Validation Study.

[10] Iron K., Zagorski B., Sykora K., Manuel D. (2008). Living and Dying in Ontario: An Opportunity to Improve Health Information.

[11] Matheson F., Dunn J., Smith K., Moineddin R., Glazier R. (2011). Ontario Marginalization Index (ON-Marg): User Guide.

[12] Elixhauser A., Steiner C., Harris D.R., Coffey R.M. (1998). Comorbidity Measures for Use with Administrative Data. Med. Care.

[13] Hall S.F. (2006). A user’s guide to selecting a comorbidity index for clinical research. J. Clin. Epidemiol..

[14] Fernandes K.A., Sutradhar R., Borkhoff C.M., Baxter N., Lofters A., Rabeneck L., Tinmouth J., Paszat L., Ontario Cancer Screening Research Network (2015). Small-area variation in screening for cancer, glucose and cholesterol in Ontario: A cross-sectional study. CMAJ Open.

[15] Beveridge R. (1998). The Canadian Triage and Acuity Scale: A new and critical element in health care reform. Canadian Association of Emergency Physicians. J. Emerg. Med..

[16] Sutradhar R., Agha M.M., Pole J., Greenberg M., Guttmann A., Hodgson D.C., Nathan P.C. (2015). Specialized survivor clinic attendance is associated with decreased rates of emergency department visits in adult survivors of childhood cancer. Cancer.

[17] Boyle L.I., Boyle A., Jay S., Marnewick J. (2020). COVID-19 lockdown impact on common general surgical acute presentations to a regional centre in New Zealand. N. Z. Med. J..

[18] McLean R.C., Young J., Musbahi A., Lee J.X., Hidayat H., Abdalla N., Chowdhury S., Baker E.A., Etherson K.J. (2020). A single-centre observational cohort study to evaluate volume and severity of emergency general surgery admissions during the COVID-19 pandemic: Is there a “lockdown” effect?. Int. J. Surg..

[19] Metelmann I.B., Busemann A. (2020). Elective surgery in times of COVID-19: A two-centre analysis of postponed operations and disease-related morbidity and mortality. Z. Evid. Fortbild. Qual. Gesundh. Wesen.

[20] Knisely A., Zhou Z.N., Wu J., Huang Y., Holcomb K., Melamed A., Advincula A.P., Lalwani A., Khoury-Collado F., Tergas A.I. (2021). Perioperative Morbidity and Mortality of Patients With COVID-19 Who Undergo Urgent and Emergent Surgical Procedures. Ann. Surg..

[21] Hakim A.A., Kellish A.S., Atabek U., Spitz F.R., Hong Y.K. (2020). Implications for the use of telehealth in surgical patients during the COVID-19 pandemic. Am. J. Surg..

